# Editorial: Emotions and Cognition in Financial Decision-Making

**DOI:** 10.3389/fpsyg.2021.811243

**Published:** 2021-12-10

**Authors:** Neal Stuart Hinvest, Richard Fairchild, Lucy Ackert

**Affiliations:** ^1^Department of Psychology, University of Bath, Bath, United Kingdom; ^2^School of Management, University of Bath, Bath, United Kingdom; ^3^Department of Economics, Finance, and Quantitative Analysis, Kennesaw State University, Kennesaw, GA, United States

**Keywords:** emotion, cognition, decision making, behavioral finance, behavioral economics, neuroeconomics

The relationship between emotions and cognition and their impact upon behavior has moved through several trends, beginning with theory positing that “hot” emotions are simply a by-product of “cold” cognition through to the widely accepted perspective that emotions are an integrative signal within decision-making and that cognitions and emotions influence each other to drive behavior. It is within this integrative perspective that behavioral finance aims to elucidate human factors within the marketplace, and wherein lies this Research Topic. A reliance on theory and methods that ignore or avoid human emotional influences on decision making under uncertainty has been put forward for, at least partial, blame in several significant real world debacles including stock market bubbles and crashes, questionably immoral organizational culture and significant mismanagement of projects leading to significant reputational damage to organizations and in some cases, their complete downfall.

Behavioral finance aims to produce predictive models of human behavior within financial contexts, leading to a step-change in the understanding of human factors in such contexts. To achieve this goal, human factors that play significant roles in financial decision making need to be identified and their impacts on observable behavior measured. Through such effort, researchers can provide information for subsequent modeling and the creation of efficacious psychological interventions, an ambitious challenge given the breadth of human behavior to be considered. This Research Topic brings together a range of modern research using a variety of empirical methods under this one aim.

A key concept within the understanding of human factors in financial decision-making is how humans perceive and represent risk. Risk is ubiquitous within financial decisions (indeed, almost all decisions we make) given that cost-benefit analyses are typically involved which entail some form of estimated risk. Liu et al., in their paper *Influence of the manner of information presentation on risky choice* show that presenting well-defined lotteries either by *alternatives* (presenting each alternative in turn) or by *dimensions* (presenting either the two magnitudes or two probabilities of each choice together) is associated with differences in utilization of expected utility calculations between presentation frames and that this effect is affected by the amount of covert attention to the presentation of information. Wang et al., in their paper *Influence factors for decision-making performance of suicide attempters and suicide ideators: the roles of somatic markers and explicit knowledge* find evidence to suggest that suicide attempters, a relatively less-researched population, use a compensatory choice strategy on the Iowa Gambling Task to improve performance to the level of healthy controls. Another common factor in every-day decision making is the need to wait for outcomes to be experienced. Yang et al. investigate how the detail in which a delay is described or cut into segments affects delay discounting in their paper *Time unpacking effect on inter-temporal decision-making: Does the effect change with choice valance?* They find that time unpacking influences tolerance of delay, regardless of whether the unpacking is of positive, neutral or negative valence (for example, receiving a gift, washing hair or attending a funeral, respectively).

Neuroeconomics is the relatively new science building on the integration of economics and psychology within behavioral economics to include methods and understanding from cognitive neuroscience, with the aim of building predictive models of human behavior considering the structure and function of the human brain, in other words, building models fitted to how the brain works. Hinvest et al. investigate the relationship between anticipatory emotions and trading performance using a complex trading simulation in their paper *Do emotions benefit investment decisions? Anticipatory emotions and investment decisions in non-professional investors*. The Authors measure galvanic skin response to find that emotions integrated into decision-making are neither wholly beneficial nor detrimental to trading performance but that the relationship is dependent on the behavior of the stock market. Gao et al. in their paper *More negative FRN from stopping searches too later that too early? An ERP study* measure Feedback Related Negativity (FRN). They report that this neural marker of feedback-driven attentional signaling is greater when a participant spends too long on information search vs. when they spend too little time, positing that this is indicative of the level of experienced regret. Suo et al. measure the carry-over effects of anger and sadness on inter-temporal choice in their paper *The differential effects of anger and sadness on intertemporal choice: an ERP study*. The Authors find that facial anger primes affect discounting behavior and peak of particular event-related potentials (ERPs), subsequently suggesting psychological factors underlying the changes. Guttman et al. take a structural approach to the brain in their paper *Age influences loss aversion through effects on cortical thickness*, finding that atrophy of the posterior cingulate cortex after middle adulthood is associated with decreases in loss aversion. Yang et al. mix neuroeconomics with a popular contemporary topic, that of social investing, in their paper *Are people altruistic when making socially responsible investments? Evidence from a tDCS study*. Via the use of transcranial direct current stimulation (tDCS), the Authors modulated the activity with the right temporo-parietal junction, an area previously associated with altruism and social behavior, and find that this influences willingness to invest in a more socially-minded way.

Finally, Bossaerts provides an intriguing review entitled *How neurobiology elucidates the role of emotions in financial decision-making*. In the review, Bossaerts uses a recently published paper with a relatively small sample size as an example to elucidate misconceptions around the integration of emotion into risk-based decision-making, highlighting the value that cognitive neuroscience can bring to traditional economic models of behavior.

This Research Topic beings together a range of methods and perspectives to elucidate human factors in financial decision-making. This is befitting to the field which crosses many disciplines and engages a wide range of academic and practitioner stakeholders. In traditional financial research, the outcome of the decision-making process is focal. While some classical theory incorporates a role for emotion, the contributions in this Research Topic illustrate the interaction between emotion and cognition. Both emotion and cognition should be recognized as fundamental components and dual forces in understanding financial decision-making.

## Author Contributions

All authors listed have made a substantial, direct, and intellectual contribution to the work and approved it for publication.

## Conflict of Interest

The authors declare that the research was conducted in the absence of any commercial or financial relationships that could be construed as a potential conflict of interest.

## Publisher's Note

All claims expressed in this article are solely those of the authors and do not necessarily represent those of their affiliated organizations, or those of the publisher, the editors and the reviewers. Any product that may be evaluated in this article, or claim that may be made by its manufacturer, is not guaranteed or endorsed by the publisher.

